# Sonication of Yeast Biomasses to Improve the Ageing on Lees Technique in Red Wines

**DOI:** 10.3390/molecules24030635

**Published:** 2019-02-12

**Authors:** Juan Manuel del Fresno, Antonio Morata, Carlos Escott, Iris Loira, Rafael Cuerda, José Antonio Suárez-Lepe

**Affiliations:** 1 enotecUPM, Chemistry and Food Technology Department, Escuela Técnica Superior de Ingeniería Agronómica, Alimentaria y de Biosistemas, Universidad Politécnica de Madrid, 28040 Madrid, Spain; juan.fresno.florez@alumnos.upm.es (J.M.d.F.); carlos.escott@gmail.com (C.E.); iris.loira@upm.es (I.L.); joseantonio.suarez.lepe@upm.es (J.A.S.-L.); 2 Comenge Bodegas y Viñedos SA, Curiel de Duero, 47316 Valladolid, Spain; cuerda@comenge.com

**Keywords:** ultrasound (US), sonicated lees, ageing on lees (AOL), polysaccharides, dissolved oxygen, phenolics, volatiles, oak wood chips, sensory profile

## Abstract

Ageing on Lees (AOL) is a technique to improve the aromatic and gustatory complexity of wine, mainly by improving its body and reducing its astringency. However, the autolytic process is slow, resulting in high production costs. This work evaluated the effect of adding sonicated lees and combining it with oak chips, as a new technique to accelerate the AOL process and improve the aromatic quality of aged red wine. Cell disruption due to sonication was verified by optical microscopy. Volatile acidity, total polyphenol index, color intensity, tonality, dissolved oxygen, anthocyanins, and fermentative volatiles were monitored throughout the ageing of the wines. Sensory analysis was performed at the end of the ageing process. Polysaccharides released from the cell walls and the oxygen consumption, was quantified using a hydroalcoholic solution. The results indicated a 20% increase of the polysaccharide content and suggested an increase in the antioxidant capacity of the lees. No significant changes were observed in the fermentative volatile compounds and the total polyphenol index (TPI), except for those wines in contact with wood. The sonication of lees had some protective effect on the total anthocyanins content, however, color intensity was significantly lower in the sonicated treatments. The sonication of the lees did not cause any defect at the sensory level. Therefore, sonication could allow a reduction in the SO_2_ addition to wine, as well as a shortening of the ageing times.

## 1. Introduction

Ageing on lees (AOL) is a technique traditionally used in the production of sparkling wines [1]. It is also used in the production of red wine, and has been extended across all winemaking areas [2]. Significant changes in the composition of wine may occur as a result of the AOL process; these changes are due to the autolysis of yeast, during the ageing [3]. The cellular autolysis of yeast involves the transfer of certain compounds to wine, such as cellular proteins, nucleic acids, lipids, and polysaccharides. Of these compounds, the polysaccharides have an effect on the physico-chemical properties of the wine, as well as in the sensory properties [4]. Some of the main effects of AOL on red wines include, oxygen consumption by the lees [5], the transfer of volatile compounds that provide complexity and persistence [6], an increase in wine density as a result of the release of high molecular weight polysaccharides from the cell walls [7], and the wine oxidation protection.

AOL has a number of economic impacts because of the large infrastructure investments producers make to store the wines in cellars, and winemakers face additional potential risks, such as microbiological and organoleptic alterations [8]. It is a slow technique, in fact; in “cava” elaboration (traditional sparkling wine from Spain), the wine spends a minimum of nine months in contact with the yeast lees [3]. The use of ultrasound (US) could be an interesting technique to accelerate the yeast autolysis and, therefore, reduce the ageing time of the wines in contact with the lees.

Ultrasound is sound waves with frequencies higher than the upper audible limit of human hearing, typically ~20 kHz [9]. Ultrasound waves induce an acoustic cavitation (formation, growth, and implosive collapse of bubbles). This phenomenon generates different chemical reactions; in addition, it can accelerate reaction rates [10]. The application of US in food technology is well documented [11]. Concerning the field of oenology, several applications have been studied, including the extraction of aroma compounds in white wine [12], the extraction of anthocyanins and other phenolic compounds from wine lees [13,14], the increased extraction of phenolic compounds from oak chips [6], and the acceleration of wine ageing [15,16]. Some authors have investigated the application of US in the AOL process [8,17]. The use of ultrasound (US) could be an alternative technique to accelerate yeast autolysis, and therefore, reduce the contact time with the lees.

In previous studies by the authors, the polysaccharide content of red wines increased after the use of US, during AOL [18]. However, the total anthocyanins content and the fermentative volatile compounds were negatively affected by the US treatment. In this study, we evaluated the use of sonicated lees—prior to its addition to the wines—to accelerate the AOL process without causing any detrimental effects, by sonicating the red wines.

## 2. Results and Discussion

### 2.1. Effects of Ultrasound Treatment on Wet Lees: Polysaccharides Released during Cellular Autolysis and Decanting Time of Hydroalcoholic Solutions

**Figure 1:**
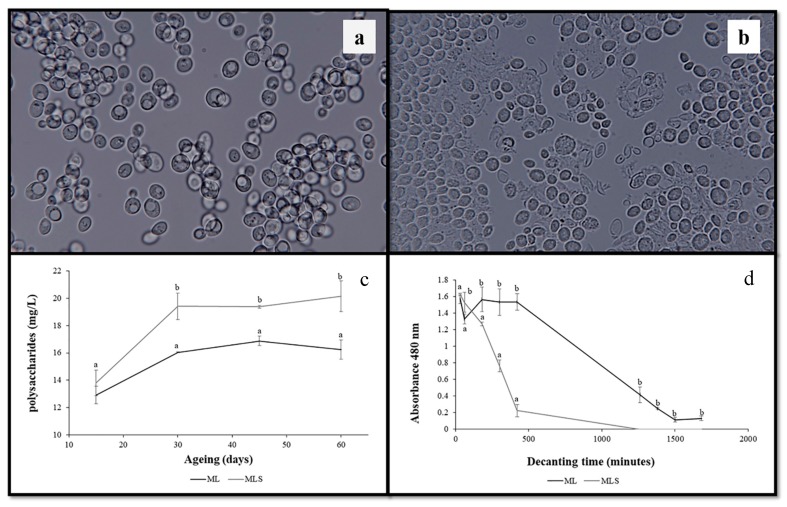
(**a**) Wet lees without ultrasound treatment; (**b**) wet lees after twenty minutes of ultrasound treatment; (**c**) evolution of the polysaccharides content (mg/L) in hydroalcoholic solutions, throughout the Ageing on Lees (AOL); (**d**) decanting time measured by spectrophotometry, at 480 nm (absorbance units). ML—hydroalcoholic solution with lees; MLS—hydroalcoholic solution with sonicated lees. Mean ± standard deviation of the three replicates. Different letters in axes X in figures (**c**) and (**d**) indicate values with statistical significant differences (*p* < 0.05).

The effect on the wet lees was observable through an optical microscope, after 20 min of US treatment (Figure 1a,b). Cell lysis effects were observed and were similar to those of cells aged for long periods of time, following traditional techniques [19].

**Figure 2:**
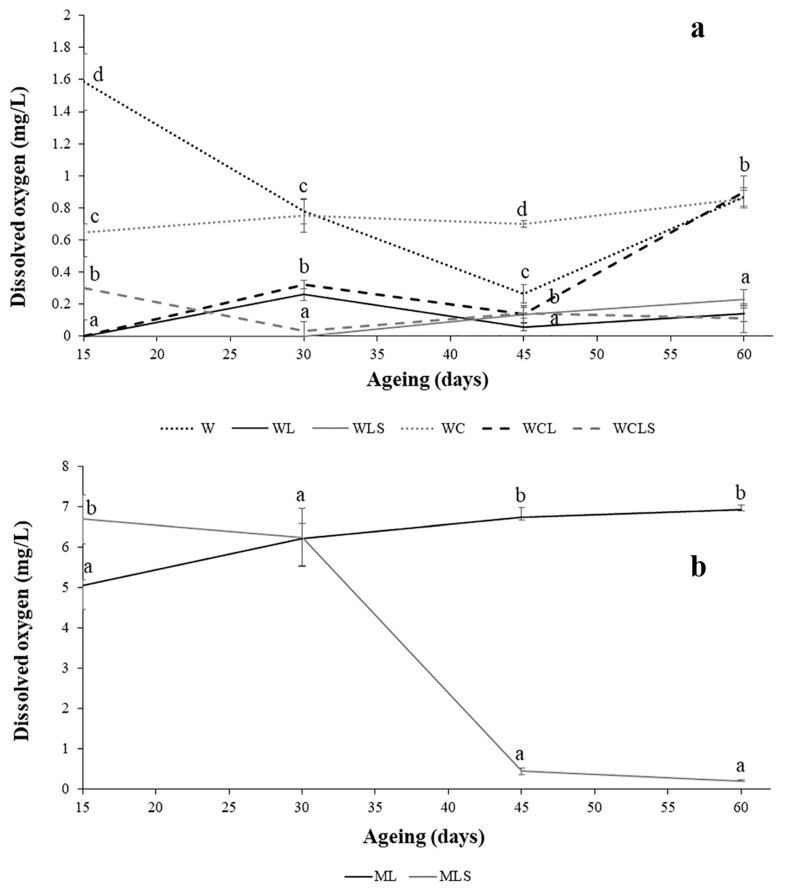
Dissolved oxygen content (mg/L) throughout the AOL, in red wines (**a**) and in the hydroalcoholic solution (**b**). W—control wine; WL—wine with lees; WLS—wine with sonicated lees; WC—wine with oak chips; WCL—wine with lees and oak chips; WCLS—wine with sonicated lees and oak chips; ML—hydroalcoholic solution with lees; MLS—hydroalcoholic solution with sonicated lees. Mean ± standard deviation of the three replicates. Different letters in axes X indicate values with statistical significant differences (*p* < 0.05).

The lysis effect seems to be responsible for an increase in the content of polysaccharides released from the cell wall, during ageing in the hydroalcoholic solution. The polysaccharides are released by the yeast, during the AOL process, in particular, mannoproteins, which play an important role, since they may interact with volatile compounds, contribute to protein and tartrate stability, stabilize the color, and decrease both the astringency and the bitterness of the wine [20]. No significant differences could be found between samples at the beginning of the ageing process (Figure 1c). However, after 30 days, the samples aged on the sonicated lees, the hydroalcoholic solution with sonicated lees (MLS), had a higher concentration of polysaccharides than the hydroalcoholic solution with lees (ML) samples. At the end of the ageing period, the concentration of polysaccharides in these samples reached 20 mg/L on average. This may be linked to the decrease in dissolved oxygen (Figure 2b), thus, showing the antioxidant capacity of the lees.

The US treatment also had an effect on the cell decanting time in the hydroalcoholic solutions (Figure 1d). After 30 min, the MLS showed significantly low values of absorbance. These samples showed values around zero at 20.8 h of static decantation. This effect highlights the importance of the “bâtonnage” process during AOL, especially when the lees is sonicated.

### 2.2. Dissolved Oxygen throughout Ageing

The control wines (control wine (W) and wine with oak chips (WC)) showed the greatest concentration of dissolved oxygen, during all ageing periods (Figure 2a). With regards to the samples aged on lees, the non-sonicated samples (wine with lees (WL), and wine with lees and oak chips (WCL)) showed values of approximately 0.03 mg/L, after 15 days; these values increased up to 0.4 mg/L, after 30 days. The oxygen concentrations remained stable in the WL samples, but increased in the WCL samples, reaching similar values to those found in the control wines (approx. 1 mg/L). A slight increase in the dissolved oxygen concentrations of the sonicated samples was evident but remained constant, throughout the ageing process. US treatment could increase the antioxidant capacity of the wine. No significant differences were found between the samples aged with oak chips and those aged without them.

In general, lower concentrations of dissolved oxygen were found in wines, compared to the hydroalcoholic solutions (Figure 2b). This could be due to the presence of several antioxidant compounds in red wines, such as polyphenols [21]. After 15 days, ML samples showed less dissolved oxygen than the MLS samples. However, after 30 days, this tendency changed completely, and the values increased, until no significant differences between the two treatments, were found. It is important to note that, at the end of the ageing period, MLS had low concentrations of dissolved oxygen (approx. 0.3 mg/L). It appears that more antioxidants from the yeast cell wall were released in the sonicated lees. Proteins and glucans are considered the main fractions responsible for the yeast cell wall antioxidant activity; in particular, thiol groups from denatured proteins [22]. The antioxidant effect of the sonicated lees was not clearly identified in the wines, perhaps due to the matrix effect of the wine; especially, the interaction with other antioxidants, such as polyphenols.

### 2.3. Volatile Acidity, Total Polyphenol Index, and Chromatic Characteristics, throughout Ageing

Regarding volatile acidity, no significant differences were found between the samples during the first 30 days of ageing (0.38 ± 0.01 g/L); after 60 days, differences were found between the wine with sonicated lees and oak chips (WCLS) and the wine with sonicated lees (WLS), with a slight increase in the WCL samples (0.44 ± 0.01 g/L). There was no noticeable increase in the volatile acidity associated with the use of lees, sonicated lees, or oak chips.

**Figure 3:**
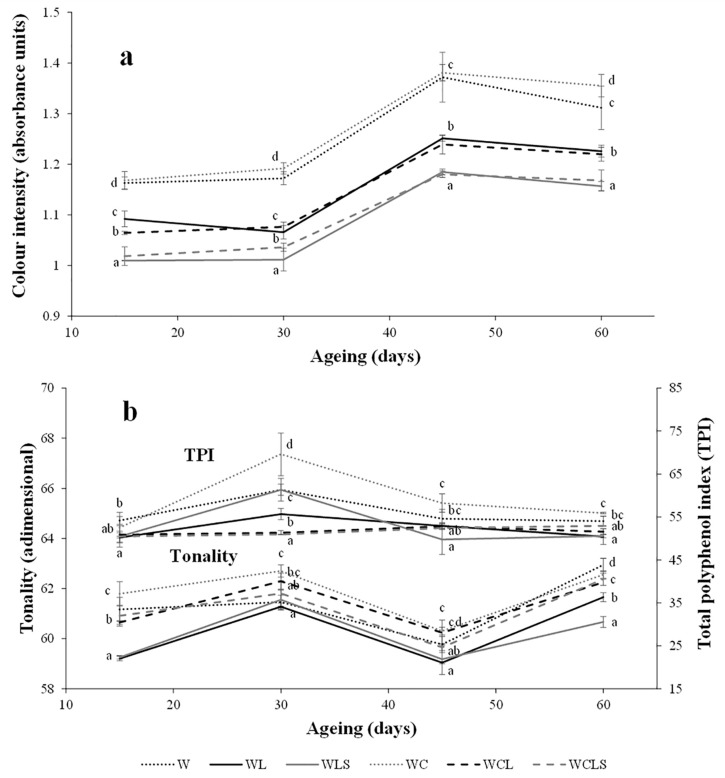
Evolution of color intensity (**a**), and tonality and total polyphenol index (**b**), during the ageing on lees. W—control wine; WL—wine with lees; WLS—wine with sonicated lees; WC—wine with oak chips; WCL—wine with lees and oak chips; WCLS—wine with sonicated lees and oak chips. Mean ± standard deviation of three replicates. Different letters in axes X indicate values with statistical significant differences (*p* < 0.05).

An increase in color intensity was evident for all samples, in the last 30 days of the ageing period (Figure 3a). The color intensity was greater in the control wines (W and WC), especially in the WC samples. Other authors also found an increase in the color intensity in the control wines, as compared to the wines aged on lees [4]. The sonication of the lees was associated with a decrease of the color intensity, while the presence of oak chips was not linked to this chromatic parameter. With respect to tonality (Figure 3b), after 15 days of ageing, the highest values of this parameter were obtained in the control wines (W and WC), while the wines aged on the lees and oak chips showed intermediate values (WCL and WCLS), and the wines aged only on lees had the lowest values (WL and WLS). Similar results were obtained at the end of the ageing period. The sonication of lees was, therefore, not related to this parameter, unlike the presence of oak chips, which seemed to increase the tonality.

Regarding the total polyphenol index (TPI) (Figure 3b), significant differences were found only between the control wines (W and WC) and the wines aged on lees (WL and WLS), after 60 days of ageing. These results are similar to those reported by Mazauric & Salmon [23], which showed a TPI decrease after one week of AOL. It is probably due to the adsorption of polyphenols by the yeast cell wall. In the case of the WCL and the WCLS samples, this effect might be compensated by the release of the polyphenols from the oak chips.

### 2.4. Evolution of Anthocyanins during Ageing

**Figure 4:**
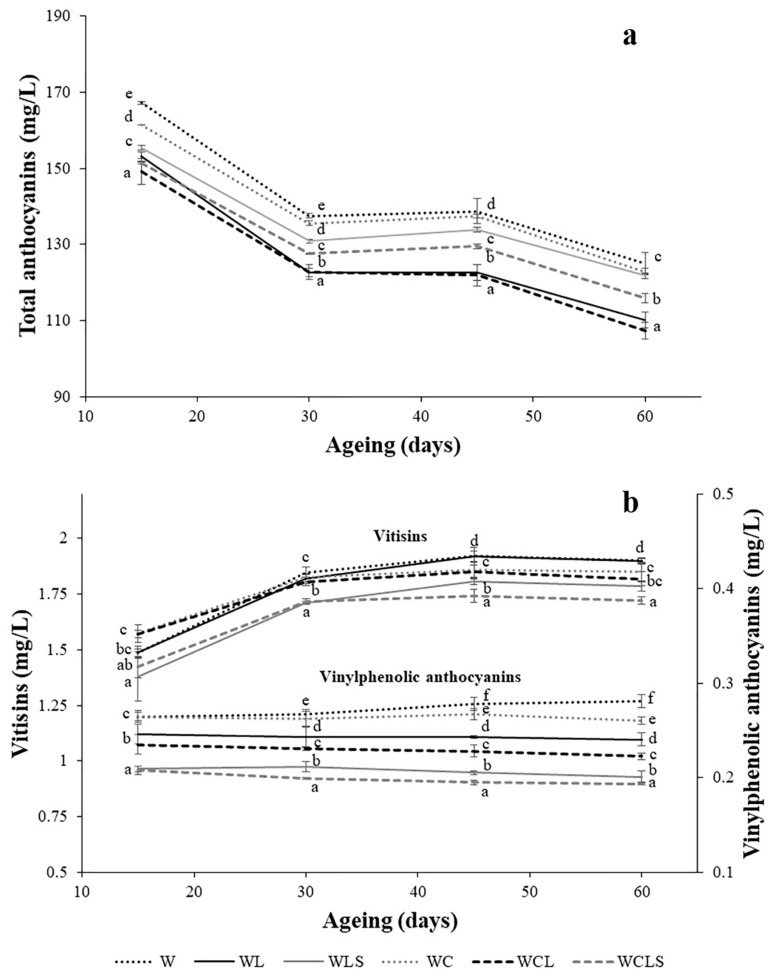
Total anthocyanins (mg/L) (**a**), vinylphenolic anthocyanins (mg/L) and vitisin content (mg/L) (**b**), throughout the AOL, in red wines. W—control wine; WL—wine with lees; WLS—wine with sonicated lees; WC—wine with oak chips; WCL—wine with lees and oak chips; WCLS—wine with sonicated lees and oak chips; ML—hydroalcoholic solution with lees; MLS—hydroalcoholic solution with sonicated lees. Mean ± standard deviation of three replicates. Different letters in axes X indicate values with statistical significant differences (*p* < 0.05).

Figure 4a shows the evolution of the total anthocyanin content. All wines showed a decrease in anthocyanins throughout the ageing period. Those wines aged on lees revealed an adsorbent effect on the anthocyanins, in comparison to the control samples (W and WC); this effect has already been reported on by other authors [24]. However, the sonication of lees (WLS and WCLS) had a protective effect, possibly due to the fact that the adsorption of pigments was greater when the cell wall was unbroken. The presence of oak chips increased the pigment loss when the lees was sonicated (WCLS).

Stable pigments were formed because of the condensation between grape anthocyanins and the metabolites, acetaldehyde and pyruvic acid [25]. At the end of the ageing period, all wine samples had an increase in the vitisin content (Figure 4b); this increase was greater in the control wines (W and WC) and in the WL wines. The sonication of lees resulted in a loss of vitisins, in wines aged in the presence of oak chips (WCLS), as well as in the samples without chips (WLS). In addition, the presence of chips (WC, WCL and WCLS) resulted in lower vitisin values, compared to the controls (W, WL, and WCL). This could perhaps be due to a greater adsorption of the vitisins by the oak chips or due to a greater interaction with compounds extracted from the wood. More specific studies would be necessary to clarify it.

As a result of the condensation between the anthocyanins and the vinylphenols, another stable pigment was formed, i.e., vinylphenolic pyranoanthocyanin [25]. In general, all wine samples showed little variations of vinylphenolic pyranoanthocyanins, throughout the ageing period, with values between 0.2 and 0.3 mg/L (Figure 4b). Nevertheless, significant differences were found at the end of the ageing period. The control wine samples (W and WC) had the highest concentrations of these pigments. Comparatively, there was an increase in the loss of these stable pigments, when the wines were aged with the sonicated lees. In the same way, the presence of the oak chips seemed to contribute to this loss. Similar to what was suggested for vitisins, a greater interaction with phenolic compounds from wood might be responsible for this evolution in the concentrations of vinylphenolics.

### 2.5. Evolution of Fermentative Volatile Compounds during Ageing

**Figure 5:**
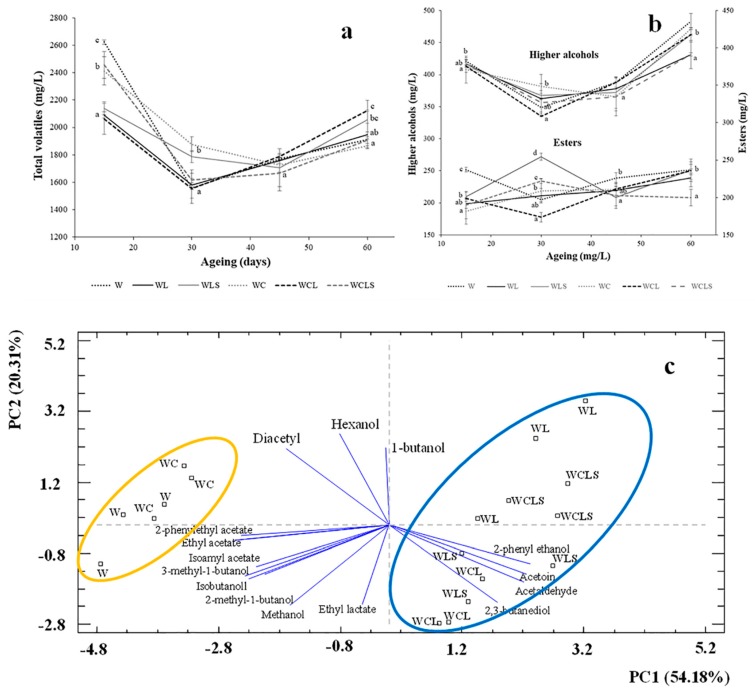
(**a**) Evolution of fermentative volatile compounds (mg/L) and (**b**) increase in higher alcohols and evolution of total ester compounds. (**c**) Principal component analysis (PCA) of the volatile compounds. Mean ± standard deviation of three replicates. W—control wine; WL—wine with lees; WLS—wine with sonicated lees; WC—wine with oak chips; WCL—wine with lees and oak chips; WCLS—wine with sonicated lees and oak chips. Different letters in axes X in figures (**a**) and (**b**) indicate values with statistical significant differences (*p* < 0.05).

During the first 30 days of ageing, the control wines (W and WC) showed significantly higher total volatile content than the wines aged on lees (WL and WCL) (Figure 5a). In addition, no significant differences were found when the lees were sonicated (WLS), with the exception of those wines aged in the presence of oak chips (WCLS). In the latter case, a significant increase of total volatiles was found, with respect to the control wines (WCL). As the ultrasounds were applied on the lees, but not on the wine, its aromatic profile was not damaged. However, at the end of the ageing period, the sonication treatment of the lees resulted in a significant difference between the WCL and the WCLS wines. Therefore, we could not determine whether the sonication treatment increased the release of total volatiles from the lees.

After 60 days of ageing, an increase in the higher alcohols content (>400 mg/L) was found in all of the wines. These values are seen as a negative quality factor [26]. Significant differences were only found between the WL and the WCLS wines (Figure 5b).

Figure 5b also illustrates the evolution of the total ester compounds, throughout the ageing period. After 30 days, the concentrations of these compounds were significantly higher in wines aged in the presence of the sonicated lees (WLS and WCLS), compared to the controls (WL and WCL). However, at the end of the ageing period, all wines had concentrations of approximately 230 mg/L, with the exception of the WCLS wines, which had significantly low concentrations.

A PCA was carried out for the 16 volatile compounds (Figure 5c). The distribution is explained by the first two principal components. PC1 is positively associated with 2-phenyl ethanol, acetaldehyde, 2,3-butanediol, and acetoin. A cluster that includes wines aged on lees could be identified on the positive value of PC1, as the concentration of these volatiles were high; on the contrary, on the negative values of the principal component PC1, a cluster composed of the control wines was found. It is interesting to note that PC1 and PC2 could not separate the wines aged on the lees and the wines aged on the sonicated lees.

### 2.6. Sensory Analysis

**Figure 6:**
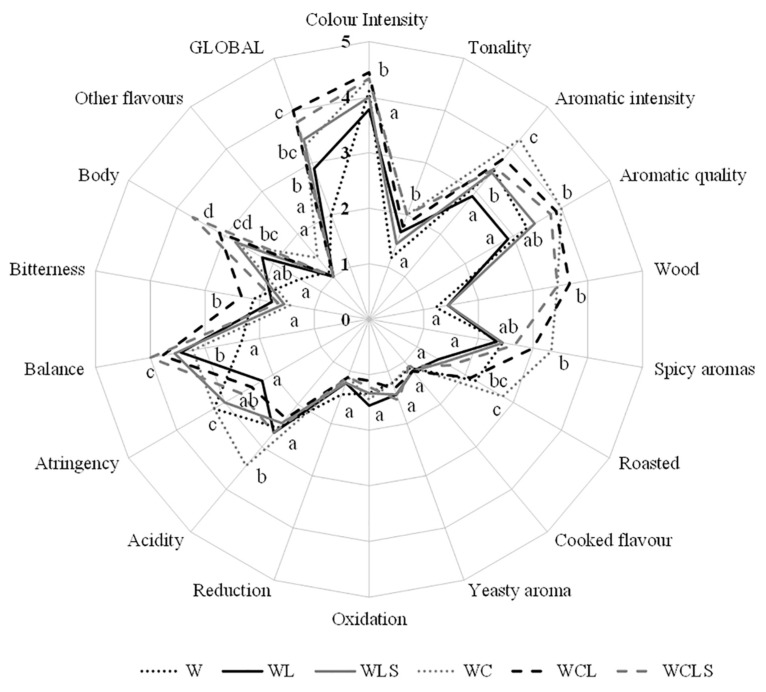
Sensory analysis carried out by a panel of nine experienced tasters. W—control wine; WL—wine with lees; WLS—wine with sonicated lees; WC—wine with oak chips; WCL—wine with lees and oak chips; WCLS—wine with sonicated lees and oak chips. Different letters in the same axis indicate values with statistical significant differences (*p* < 0.05).

The wines aged with oak chips and lees obtained the highest scores in terms of global quality, possibly because of the aromatic complexity, due to the combination of both ageing systems [24] (Figure 6). In the same way, tasters highlighted the control wine as the lowest in global quality. The WL wines showed significant differences with the lowest scores in color intensity. This characteristic was not confirmed by spectrophotometric analysis. The WC and WCLS wines were significantly high in tonality. In terms of the parameters evaluated in the aromatic phase, the tasters identified the wines aged with oak chips as those with the highest aromatic intensity. In addition, these wines obtained the highest score for aromatic quality. The tasters positively assessed the contribution of the wood volatiles to the complexity of the wines. None of the wines tasted had aromatic defects associated with oxidation or reduction. It is also interesting to note that the yeast aroma was not detected either. There were significant differences of acidity in the WC wines. Moreover, the W and the WC wines obtained the highest scores for the astringency parameter. These results are in accordance with the highest values of Total Polyphenol Index (IPT) analyzed by the spectrophotometric techniques. The scores for “body” were statistically significantly highest in the WCLS and the WCL wines. It can be concluded that sonication of the lees, prior to its addition to the wine, had no additional negative effects on the sensory profile of the wines, beyond those changes related to a traditional ageing on lees.

## 3. Materials and Methods

### 3.1. Yeast Lees Biomass

The yeast strain CTPL22 (*Saccharomyces cerevisiae*) used in this study was isolated in “Comenge Bodegas y Viñedos SA” by the Chemistry and Food Technology Department of ETSI Agronómica, Alimentaria y de Biosistemas (UPM). This winery is situated in the Ribera Del Duero area of Spain.

The yeast lees biomass was obtained by growing in 2 L of YEPD medium with 100 g/L of glucose. The growth was carried out at 25 °C, with orbital shaking for 3 days. After this, the biomass was washed three times with deionized water, discarding the supernatant after each centrifugation, at 1200 rcf, for 3 min.

### 3.2. Red Wine and Hydroalcoholic Solution

The red wine used in this experiment was elaborated by Bodegas Comenge (Ribera del Duero, Spain), conventionally produced with grapes from the 2016 harvest (*Vitis vinifera* L. cv. Tempranillo). The wine was aged for 3 months in French oak barrels. The following parameters were measured: pH = 3.6; volatile acidity = 0.38 g/L of acetic acid; ethanol content = 13.5% *v*/*v*; lactic acid = 2 g/L; total SO_2_ = 80 mg/L; total polyphenol index (TPI) = 56; total anthocyanin content = 175 mg/L; color intensity = 1.2 absorbance units; tonality = 60.8 (adimensional); and total fermentative volatile compounds = 1755.8 mg/L (see the analytical methods described below).

A hydroalcoholic solution composed of water and ethanol (13.5% *v*/*v*) was used to simulate the AOL in the red wine and measure the content of polysaccharides released by the lees. In addition, this hydroalcoholic solution was sulphited to 80 mg/L of the total SO_2_ with K_2_S_2_O_5_. The pH was adjusted to 3.6, with phosphoric acid.

### 3.3. Ultrasound Treatment

50 mL of pure yeast biomass was treated with ultrasound, for twenty minutes, in a 100 mL volume ISO flask. A UP400ST digital ultrasonic processor (Hielscher, Teltow, Germany) of 400 W and 24 kHz, equipped with a H22DL2 sonotrode (Ø = 22 mm, submerged depth = 165 mm, max. amplitude = 50 µm and acoustic power density = 52 W/cm^2^), was used to apply the US treatment. The sonotrode was introduced into the yeast biomass until the headspace was completely filled. The US treatment was sufficient when the cellular lysis effects were evident using an optical microscope.

### 3.4. Samples Preparation

The wine samples were prepared in triplicates, using ISO flasks of 1 L. The different treatments were: the control wine (W), wine with lees (WL), wine with sonicated lees (WLS), wine with oak chips (WC), wine with lees and oak chips (WCL), and wine with sonicated lees and oak chips (WCLS). The dosage of oak chips was 2.5 g/L. The medium-toasted oak chips were provided by Boisé (Villeneuve-lès-Maguelone, France). The dosage of wet lees was 6 g/L. The wine samples were aged in a dark room, at a temperature of 14 °C, for 60 days. The samples were shaken manually, once a week, without opening the flasks, with the aim of resuspending the lees in the wine and, thus, increasing the contact surface. Samples were drawn every two weeks to measure the dissolved oxygen, volatile acidity, TPI, chromatic characteristics (color intensity and tonality), fermentative volatile compounds, and the anthocyanin content. A sensory analysis was performed at the end of the ageing period.

The hydroalcoholic solutions were also prepared in triplicates, using ISO flasks of 1 L. The different treatments were: hydroalcoholic solution with lees (ML) and hydroalcoholic solution with sonicated lees (MLS). The dosage of wet lees was 6 g/L. The ageing conditions were the same as those of the wine samples. Every two weeks, the analyses undertaken were dissolved oxygen and polysaccharides content. Absorbance at 480 nm was monitored after a stirring cycle, in order to establish the differences in the decanting time of the lees (25 °C).

### 3.5. Polysaccharides Analysis (HPLC-RI)

The analysis of polysaccharides was only done in the hydroalcoholic solutions, using an HPLC-RI technique. A Waters e2695 (Waters, Milford, MA, USA) chromatograph with a Ultrahydrogel 250 molecular exclusion column (Waters) was used, according to the method described by Loira and co-workers [24].

### 3.6. Volatile Acidity and Spectrophotometric Analyses

The volatile acidity (expressed as acetic acid) was measured by Fourier transform infrared spectroscopy (FTIR), using an OenoFoss™ instrument (FOSS Iberia, Barcelona, Spain). The decanting time was obtained by consecutive measurements of absorbance at 480 nm, for a total time of 28.33 h in the hydroalcoholic solutions, at the end of the ageing period. The TPI of the wine samples was calculated by spectrophotometric measurements, after diluting the samples 100 times with deionized water (absorbance at 280 nm with 1 cm path length quartz cuvette) [27]. The color intensity and the tonality were also measured spectrophotometrically, but without diluting the samples (absorbance at 420, 520, and 620 nm with 1 mm path length glass cuvette) [28]. All spectrometric measurements were obtained using an 8453 spectrophotometer from Agilent Technologies™ (Palo Alto, CA, USA).

### 3.7. Analysis of Dissolved Oxygen

A HI9146V oximeter (Hanna instruments, Woonsocket, RI, USA) was used to determine the dissolved oxygen content, both in the wine samples and the hydroalcoholic solutions. A magnetic stirrer was used for homogenization around the membrane. The reading was made after 1 min, while keeping the probe inside the sample, under agitation, for the time necessary for the stabilization of the oximeter.

### 3.8. Anthocyanins and Pyranoanthocyanins

The anthocyanin and pyranoanthocyanin contents were obtained using an 1100 HPLC chromatograph (Agilent Technologies, Palo Alto, CA, USA) equipped with a diode array detector and a Kinetex RP-C18 100 Å Phenomenex column (100 × 4.6 mm; 2.6 µm) (Torrance, CA, USA), as described by Fresno and co-workers [18].

### 3.9. Fermentative Volatile Compounds

The fermentative volatile compounds were determined using an Agilent Technologies 6850 gas chromatograph, equipped with an integrated flame ionization detector (GC-FID). Analyses were performed according to the method described by Ábalos et al. [29].

### 3.10. Sensory Analysis

The sensory analysis was carried out by a panel of expert wine tasters, consisting of nine trained judges (age ranging from 28 to 50 years old; 3 women and 6 men). The blind tasting took place under fluorescent lighting and the samples were presented in a random order. Approximately 20 mL of wine was served into odor-free tasting glasses. Serving temperature was set at 20 ± 2 °C. A glass of water was used for palate cleansing between samples. The panelists used a scale from 1 (low perception) to 5 (high perception), to rate the intensity of each attribute, with the exception of the tonality parameter, which was evaluated from 1 (purple tones) to 5 (yellow tones). The visual parameters evaluated were color intensity and tonality; the aromatic parameters evaluated were aromatic intensity, aromatic quality, wood, spicy aromas, roasted, cooked flavor, yeasty aroma, oxidation, and reduction; the flavor parameters evaluated were acidity, astringency, balance, bitterness, and body. Finally, the overall impression was assessed, which included all the parameters of the wines tasted.

### 3.11. Statistical Analysis

Means and standard deviations were calculated and analysis of variance (ANOVA) and least significant difference (LSD) tests were performed using PC Statgraphics v.5 software (Graphics Software Systems, Rockville, MD, USA). The LSD test was used to detect significant differences between means. Significance was set at *p* < 0.05.

## 4. Conclusions

The use of US on wet lees could accelerate the cellular lysis, since the addition of sonicated lees to wines increased the rate of transfer of polysaccharides, during the ageing process. There seems to have been an increase in the antioxidant capacity of the lees, based on the low content of dissolved oxygen found in the wines aged with the sonicated lees. A faster decantation rate was also observed. The AOL process affected the different oenological parameters, including reductions in color intensity and TPI. In addition, when the AOL process was combined with oak chips, both aromatic intensity and aromatic quality were improved. The use of this technique in wineries could be beneficial because it might reduce the SO_2_ addition, as well as the ageing times. Furthermore, reductions in the lees decanting time could facilitate clarification, prior to bottling.
